# Reliability, Minimum Detectable Change and Construct Validity of the Functional Rating Index in Italian Patients with Chronic Non-Specific Low Back Pain

**DOI:** 10.3390/medicina62040653

**Published:** 2026-03-29

**Authors:** Teresa Paolucci, Letizia Pezzi, Andrea Pantalone, Rocco Palumbo, Roberto Di Deo Iurisci, Federico Arippa, Alice Cichelli, Ronald J. Feise, Marco Monticone

**Affiliations:** 1 Department of Oral, Medical and Biotechnological Sciences, Physical Medicine and Rehabilitation, University G. D’Annunzio, 66100 Chieti, Italy; teresa.paolucci@unich.it (T.P.); verdelu1@hotmail.it (R.D.D.I.); 2 Rehabilitation Unit, ASST Cremona—Ospedale di Cremona, 26100 Cremona, Italy; letizia.pezzi@asst-cremona.it; 3 Department of Medicine and Science of Aging, University G. D’Annunzio, 66100 Chieti, Italy; andrea.pantalone@unich.it; 4 Department of Psychological, Health, and Territorial Sciences, University G. D’Annunzio, 66100 Chieti, Italy; rocco.palumbo@unich.it; 5 Department of Mechanical, Chemical and Materials Engineering, University of Cagliari, 09141 Cagliari, Italy; federico.arippa@unica.it; 6 Institute of Evidence-Based Chiropractic, Scottsdale, AZ 85254, USA; rjf@chiroevidence.com; 7 Department of Clinical and Experimental Sciences, Università di Brescia, 24123 Brescia, Italy; marco.monticone@unibs.it

**Keywords:** pain, patient-reported outcome measures, measurement error, rehabilitation, disability

## Abstract

*Background and Objectives:* To assess the reliability and construct validity of the Functional Rating Index (FRI) in Italian-speaking individuals with chronic non-specific low back pain (CLBP), in order to improve assessment and clinical management in this population. *Materials and Methods:* This cross-sectional study consecutively enrolled 75 individuals with CLBP (52 females; mean age 48.71 ± 19.18 years; mean pain duration 298.64 ± 427.52 weeks). Internal consistency and test–retest reliability were evaluated using Cronbach’s α and the intraclass correlation coefficient [ICC_2,1_], respectively, while measurement error was estimated through the minimum detectable change (MDC). Construct validity was examined by testing a priori hypotheses through correlations (Pearson’s r) between the FRI and disability measures (Roland–Morris Disability Questionnaire, RMQ; Oswestry Disability Index, ODI), pain intensity (Numerical Rating Scale, NRS), and quality of life (Short-Form Health Survey, SF-36). *Results*: Cronbach’s α was 0.88, and test–retest reliability showed an ICC_2,1_ of 0.86 (95%CI: 0.82–0.93). The MDC was 18.05, corresponding to approximately 20% of the total score. The Italian FRI demonstrated strong correlations with the RMQ (r = 0.70) and ODI (r = 0.77), and a moderate correlation with the NRS (r = 0.60). The physical and social domains of the SF-36 showed stronger negative correlations with the FRI than the mental and emotional domains. *Conclusions*: The Italian version of the FRI is a reliable and valid instrument for individuals with CLBP and is recommended for both clinical practice and research applications.

## 1. Introduction

Low back pain is one of the most prevalent musculoskeletal conditions worldwide, with a high risk of recurrence and chronicity. Within one year, up to 70% of individuals with low back pain experience a recurrence [1]. Chronic non-specific low back pain (CLBP) is associated with increased healthcare costs, work absenteeism, and a substantial negative impact on health-related quality of life (Qol) [2,3,4].

According to recent Global Burden of Disease data, low back pain remains the leading cause of years lived with disability worldwide, representing a major public health and socioeconomic challenge. The chronic form, in particular, contributes disproportionately to long-term functional limitation and rehabilitation demand, emphasizing the need for accurate and reliable outcome assessment tools in clinical practice [5].

The assessment of pain-related disability and functional status using validated patient-reported outcome measures (PROMs) is essential for monitoring treatment effects and rehabilitation outcomes in individuals with CLBP [1]. PROMs provide clinically relevant information by capturing patients’ perceptions of activity limitations and participation restrictions, often with greater sensitivity than objective clinical indicators [1].

PROMs provide clinically relevant information by capturing patients’ perceptions of activity limitations and participation restrictions, offering a complementary perspective to objective clinical assessments in the evaluation of functional status [6].

In this context, Feise and Menke developed the Functional Rating Index (FRI), a PROM designed to assess functional limitations and symptoms related to spinal disorders [7]. The FRI is characterized by a concise structure, simple wording, and ease of scoring, making it suitable for both clinical and research settings [7]. This is significant because longer PROMs often require more time for individuals and healthcare staff to complete. However, this should not deter the use of these outcome measures, as individuals’ perceptions are crucial for shaping therapy and evaluating treatment at various time points. Indeed, PROMs may be more precise than most physiological or clinical indicators that physicians typically rely on [8].

Over the past two decades, the psychometric properties of the FRI have been extensively investigated in English-speaking and non–English-speaking populations with spinal disorders. Previous studies have consistently demonstrated good internal consistency, test–retest reliability, construct validity, and responsiveness [7]. Subsequent research has confirmed the test–retest reliability, construct validity, and responsiveness of the FRI [8,9,10].

Furthermore, the FRI has been successfully translated, culturally adapted and validated in various languages and cultures, including Korean in neck pain (NP) [11], as well as Turkish [12], Brazilian-Portuguese [13], Persian [14], Chinese [15], Iranian [16], Spanish [17] and Arabic in low back pain [18].

In Italy, the FRI was cross-cultural adapted and validated by analyzing internal consistency, test–retest reliability, and construct validity in people with neck pain [19]. However, its measurement properties have not yet been examined in Italian-speaking individuals with CLBP.

Although the Italian version of the FRI has previously undergone cultural adaptation in individuals with neck pain, differences in pain location, functional demands, and disability patterns between neck pain and CLBP populations may influence measurement performance. Importantly, psychometric properties cannot be automatically generalized across clinical populations, even within the same language context, as differences in symptom chronicity, disability levels, and rehabilitation pathways may influence measurement performance [20]. Therefore, a specific validation process conducted in individuals with CLBP is necessary to ensure accurate interpretability and cross-cultural comparability.

In addition to reliability and construct validity, interpretability parameters such as the minimum detectable change (MDC) are crucial for clinical decision-making. While reliability reflects score stability, MDC quantifies the smallest change that can be interpreted as real and beyond measurement error. This distinction is particularly relevant in chronic conditions such as CLBP, where gradual and moderate improvements are expected during rehabilitation [20].

In line with the original developer’s recommendations and previous studies suggesting a predominantly unidimensional structure of the instrument [16,21], the present study aimed to evaluate the reliability (internal consistency and test–retest reliability), interpretability (minimum detectable change), and construct validity (hypothesis testing) of the Italian version of the FRI in individuals with CLBP.

## 2. Materials and Methods

### 2.1. Study Design and Ethical Approval

This study employed a cross-sectional design. Ethical approval was obtained from the University Institutional Review Board (Prot. No. 24007, 11 April 2024; ERB DISPuTER, Department of Psychological Sciences, Health, and Territory, University of Chieti “G. D’Annunzio”, Italy). The study was conducted in accordance with the Declaration of Helsinki [22]. The COSMIN guidelines for the selection of health measurement instruments were followed [23].

### 2.2. Participants

This research consecutively involved individuals who were present at “Sant’Annunziata” Clinical Hospital of Chieti and Cremona Hospital in Italy. Consecutive sampling was adopted to reduce potential selection bias and to enhance representativeness of the clinical population. No therapeutic interventions were initiated or modified during the 7–10 day retest interval to ensure clinical stability. Eligibility criteria included: age ≥ 18 years; diagnosis of CLBP, defined as pain lasting longer than 12 weeks without a specific underlying pathology [24]; and sufficient proficiency in the Italian language. Exclusion criteria were: acute or subacute non-specific low back pain; specific spinal conditions (e.g., deformity, fracture, or spinal stenosis); neurological disorders confirmed by imaging or medical history; systemic or rheumatological diseases; cognitive impairment; previous cerebrovascular events; recent cardiovascular stroke; and inability or unwillingness to provide written informed consent.

All participants received verbal and written information about the study objectives and procedures and provided written informed consent prior to enrolment. A physiotherapist, not involved in treatment delivery and supervised by the principal investigator, conducted all assessments. Sociodemographic and clinical data were collected during the first assessment.

### 2.3. Outcome Measures

*Functional Rating Index (FRI).* It is a PROM and comprises ten items assessing pain and functional capacity related to low back pain: two items focus on pain, while eight address activities of daily living. Each item is scored on a five-point Likert scale ranging from 0 (“never”) to 4 (“always”). The total score is obtained by summing the item scores, dividing by 40, and multiplying by 100, yielding a percentage score from 0 (no disability) to 100 (severe disability) [7].

*Roland Morris Disability Questionnaire (RMQ).* It is a self-reported questionnaire assessing disability related to low back pain. It consists of 24 items, with total scores ranging from 0 (no disability) to 24 (maximum disability) [25].

*Oswestry Disability Index (ODI).* It is a self-administered questionnaire composed of 10 sections evaluating pain and disability related to spinal disorders. Each section is scored from 0 to 5, and the total score varies from 0 (no disability) to 50 (maximum disability) [26].

*Pain Numerical Rating Scale (NRS).* The NRS is an 11-point scale ranging from 0 (no pain) to 10 (worst imaginable pain) and was used to assess current pain intensity [27].

*36-Item Short-Form Health Survey (SF-36)*. The SF-36 is a generic self-administered questionnaire measuring health-related Qol across eight domains: physical functioning, role physical, bodily pain, general health, vitality, social functioning, role emotional, and mental health. Each domain is scored from 0 to 100, with higher scores indicating better health status [28].

Validated Italian versions of all instruments were used [25,26,27,28]. During the first assessment, questionnaires were administered in the following order: FRI, RMQ, ODI, NRS, and SF-36. At the second assessment, only the FRI and NRS were administered.

### 2.4. Descriptive Statistics and Psychometric Analysis

Descriptive statistics were calculated for all variables. Floor and ceiling effects were considered present if more than 15% of participants achieved the lowest or highest possible score.

Psychometric evaluations included reliability, interpretability, and hypothesis testing.

*Reliability.* This was assessed as detailed below.

(1) Cronbach’s α was used to evaluate internal consistency (i.e., the extent to which the items of a questionnaire measure the same underlying construct and are therefore interrelated), and estimates > 0.70 were considered acceptable [20]; (2) the intraclass correlation coefficient, ICC_2,1_ (two-way random effects, single measurement), was used to assess retest stability (i.e., the degree to which an instrument produces consistent and stable scores when administered to the same individuals on two separate occasions, assuming that the underlying construct has not changed), because it allows generalization of reliability estimates beyond the specific testing occasions involved in the study [20]; the FRI was administered after seven–ten days to minimize recall bias and in the absence of any therapeutic intervention. Although no formal external anchor of clinical stability (e.g., Global Rating of Change) was used, the short retest interval and the absence of treatment modifications were adopted to reduce the likelihood of meaningful clinical change; values of 0.70–0.85 were deemed good and of >0.85 excellent [24]; and (3) the standard error of measurement (SEM) (i.e., the amount of random measurement error in an observed score) was assessed through the formula [20]:

SEM = SD ∗ √(1 − ICC_2.1_)

where SD corresponds to the standard deviation of all of the evaluations gathered during the first assessment.

*Interpretability.* It was calculated using the minimum detectable change (MDC) (i.e., the smallest change in a score that can be interpreted as a real change beyond measurement error), with the following equation:

MDC = SEM ∗ z value ∗ √2

In order to achieve a 95% confidence interval (CI) for the MDC, a z estimate of 1.96 was chosen.

*Construct validity.* This was assessed by hypothesis testing, which is a statistical method used to evaluate predefined assumptions about the relationships between variables or constructs [20]. In more detail, the FRI was expected to achieve: (1) positive large correlations with the RMQ and the ODI as they measure similar constructs; (2) positive moderate-to-strong correlations with the NRS, given that 20% of the items are related to pain; (3) negative moderate correlations with most SF-36 domains primarily reflecting physical functioning and social participation (i.e., Physical Functioning, Role Physical, Bodily Pain, General Health, Vitality, and Social Functioning), alongside weaker negative correlations with domains mainly reflecting emotional and mental aspects of Qol (i.e., Role Emotional and Mental Health). Prior to correlation analyses, assumptions for Pearson’s correlation were evaluated. The distribution of total scores was inspected using histograms, skewness and kurtosis indices, and the Shapiro–Wilk test. Scatterplots were examined to assess linear relationships between variables and to detect potential outliers. As no substantial deviations were observed, Pearson’s correlation coefficients were used. The magnitude of correlations was interpreted according to thresholds proposed for pain-related outcomes, where r ≈ 0.10 indicates a small association, r ≈ 0.30 a moderate association, and r ≥ 0.40 a large association for individual differences in pain research. These thresholds have been suggested as more appropriate for interpreting correlations involving pain-related constructs [29]. This psychometric property was deemed acceptable if at least 75% of the assumptions were accomplished [24].

### 2.5. Sample Size

A sample size of at least 50 participants has been suggested as the minimum acceptable threshold for reliability analyses in studies evaluating measurement properties of PROMs [20]. However, the final sample consisted of 75 participants, exceeding this minimum recommendation. For construct validity, the sample size was estimated assuming a minimum expected correlation coefficient of r = 0.35, with a two-tailed α = 0.05 and statistical power of 80%, resulting in a required sample of approximately 62–65 participants. Therefore, the final sample size was considered adequate to test the predefined construct validity hypotheses.Accordingly, we planned to recruit approximately 70–75 participants to account for potential missing data.

### 2.6. Software and Data Availability

We performed the analysis using SPSS v.29 package (IBM, Armonk, New York, USA). Data are not openly accessible; they can be obtained from the corresponding author after a fair invitation.

## 3. Results

### 3.1. Participant Characteristics

**Figure 1:**
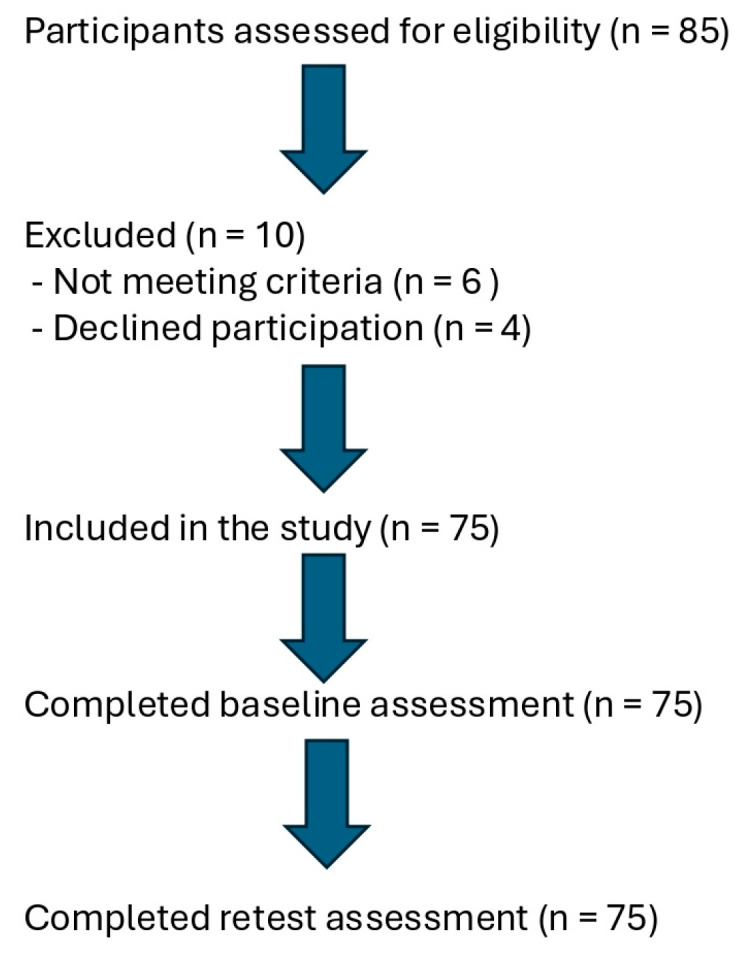
Flow diagram of study participants.

The population analyzed 52 females and 23 males, with a mean age of 48.71 ± 19.18. The average pain duration was 68.8 ± 98.4 months. Complete clinical and demographics features are stated in Table 1. The flow diagram of study participants is shown in Figure 1.

### 3.2. Descriptive Statistics and Floor/Ceiling Effects

Mean, quartiles, and floor/ceiling effects of all instruments used are retrievable in Table 2.

There were no floor/ceiling effects except for SF-36 Physical and SF-36 Emotional Role (ceiling effects of 51 and 53%, respectively).

During the fulfillment phase, the items were easily received by participants, and problems of comprehension and multiple replies did not emerge.

### 3.3. Reliability

**Figure 2:**
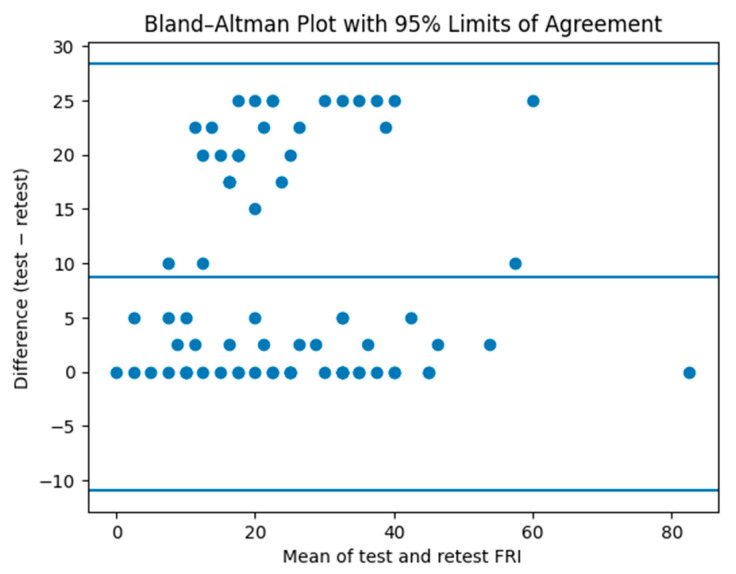
Bland–Altman plot for test–retest agreement of the Functional Rating Index. The central line represents the mean difference between test and retest scores, while the upper and lower lines indicate the 95% limits of agreement.

A strong internal consistency degree was achieved (alpha = 0.88), indicating strong interrelatedness among items within a single construct. Test–retest reliability was satisfactory (ICC_2,1_ = 0.86 [95% CI: 0.82–0.93]. The stability of clinical status over the retest interval was indirectly supported by the very high correlation between NRS scores at baseline and follow-up (r = 0.97, *p* < 0.001), suggesting minimal variation in pain intensity during the retest period. Agreement between test and retest measurements was further explored using a Bland–Altman plot (Figure 2); the mean difference between measurements was 8.7 points, with 95% limits of agreement (LOA) ranging from −10.9 to 28.4 points. The SEM was 6.53 points, indicating limited random measurement variability.

### 3.4. Interpretability

The MDC, reflecting measurement precision, was 18.05 points on the 0–100 FRI scale, suggesting that changes below this threshold should be interpreted cautiously in clinical practice.

### 3.5. Construct Validity

All predefined hypotheses for construct validity were confirmed. According to pain-specific thresholds for correlation magnitude, the FRI showed large positive correlations with the RMQ, ODI, and NRS. Regarding health-related QoL, the FRI demonstrated moderate-to-large negative correlations with SF-36 domains related to physical functioning and social participation, whereas small negative correlations were observed for domains primarily reflecting emotional and mental health (Table 3). Correlation coefficients are reported together with their 95% confidence intervals to facilitate interpretation of the strength and precision of the associations.

**Figure 3:**
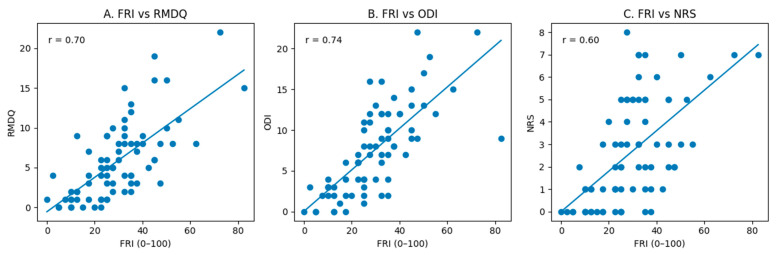
Scatterplots illustrating construct validity of the Functional Rating Index (FRI). (**A**) Relationship between Functional Rating Index (FRI) and the Roland–Morris Disability Questionnaire (RMQ); (**B**) relationship between FRI and the Oswestry Disability Index (ODI); (**C**) relationship between FRI and the Numerical Rating Scale (NRS). The solid line represents the linear regression.

Scatterplots illustrating the relationships between the FRI and disability and pain PROMs used to assess construct validity are presented in Figure 3.

## 4. Discussion

This investigation illustrates the assessment of the FRI in terms of reliability, interpretability, and hypothesis testing in Italians with CLBP.

Beyond simple replication of previous validation studies, the present findings contribute to the cross-cultural robustness of the FRI by confirming its measurement stability and construct coherence in a population characterized by chronicity and long-standing disability. CLBP represents a clinically heterogeneous condition, often associated with fluctuating symptom trajectories and multidimensional disability. Therefore, the confirmation of consistent psychometric performance in this subgroup reinforces the instrument’s applicability in complex rehabilitation contexts.

No relevant floor or ceiling effects were observed for the FRI and pain intensity measures, whereas pronounced ceiling effects emerged in the SF-36 Role Physical and Role Emotional domains, likely reflecting the binary (yes/no) response format of these subscales rather than a true absence of role-related difficulties [30].

The Functional Rating Index (FRI) displayed good internal consistency (0.88), consistent with the developers’ reported value (0.92) [7]. Similar results were observed in various adapted versions of the FRI tested in individuals with LBP: Turkish (0.92) [11], Brazilian-Portuguese (0.92) [13], Persian (0.89) [14], Chinese (0.90) [15], Iranian (0.90) [16], Spanish (0.85) [17], and Arabic (0.85) [18]. Taken together, these findings indicate a stable level of item interrelatedness across different cultural contexts, supporting the internal coherence of the FRI as a measure of pain-related functional disability.

This study demonstrated excellent agreement between retest results (ICC_2,1_ = 0.88), in line with findings from non-English validations conducted in individuals with low back pain, including Turkish (0.92) [12], Brazilian-Portuguese (0.95) [13], Persian (0.81) [14], Chinese (0.95) [15], Iranian (0.95) [16], Spanish (0.97) [17], and Arabic (0.85) [18]. The higher reliability reported in the original study (ICC_3,k_ = 0.99) [7] may partly reflect the use of a different ICC model, which estimates the reliability of averaged measurements under fixed conditions and typically produces higher values than the single-measurement ICC_2,1_ used in the present study [31]. However, differences in reliability estimates may also be influenced by several methodological factors, including sample size, variability of the study population, clinical characteristics of participants, and measurement conditions during the test–retest interval. These aspects may contribute to variations in ICC values across validation studies and should be considered when interpreting comparisons between studies. The Bland–Altman analysis showed moderate agreement between test and retest measurements, with LOA comparable to the magnitude of the MDC.

Given the observed test–retest reliability and the resulting SEM, the MDC corresponded to approximately 20% of the total score (≈19 points on the 0–100 FRI scale), indicating that score changes exceeding this threshold are unlikely to be attributable to measurement error alone. From a clinical standpoint, this threshold provides a pragmatic benchmark for clinicians and researchers when interpreting longitudinal changes. In rehabilitation settings where incremental functional gains are expected, distinguishing true recovery from measurement noise is critical for treatment adjustment, discharge planning, and outcome reporting [6]. However, the magnitude of the MDC observed in the present study (approximately 19 points) may limit the sensitivity of the instrument for detecting small but potentially meaningful individual changes over time. In the absence of established minimal important change values for the Italian version of the FRI, the clinical relevance of this threshold should therefore be interpreted with caution. Future studies should investigate MIC values to better contextualize the responsiveness of the instrument in CLBP populations. Lower MDC estimates have been reported in the Arabic version (SEM: 1.17; MDC: 3.24), likely reflecting the restriction of reliability analyses to participants classified as clinically stable using an external anchor [18], whereas stability in the present study was inferred from a short retest interval and the absence of relevant changes in pain intensity.

Construct validity was as predicted, indicating a large correlation with the RMQ and the ODI, as they are all PROMs devoted to evaluating physical functioning and investigating similar items. The original study found a good correlation (0.76) [7] with the PROM Disability Rating Scale, which, despite some items different from the FRI related to responsivity, cognitive functioning, or psychosocial adaptability, had the aim of measuring physical disability [32].

The Italian FRI was well associated with the RMQ (0.70), and large correlations were found with other cross-culturally adapted versions enrolling LBP individuals: Turkish (0.66) [12], Brazilian-Portuguese (0.80) [13], Persian (0.61) [14], Iranian (0.83) [16], Spanish (0.66) [17], and Arabic (0.62) [18]. Further, the Italian FRI was even more highly correlated with the ODI (0.74), and similar comparisons were found in the three other adapted versions investigating people with low back pain who adopted the same PROM: Persian (0.75) [14], Iranian (0.96) [16], and Arabic (0.65) [18].

The FRI was also compared with the NRS, showing a moderate correlation as pre-hypothesized (0.60). This magnitude of correlation is clinically plausible, as pain intensity represents only one component of disability, whereas the FRI primarily captures the impact of pain on daily activities and functional performance rather than pain severity alone. A similar correlation (0.66) was found in the Brazilian-Portuguese (0.67) [13], Spanish (0.66) [17], and Arabic population [18], while slightly higher correlations were pointed out in the other adapted versions of the FRI involving LBP people: Turkish (0.70) [12], Persian (0.73) [14], Chinese (0.85) [15], and Iranian (0.72) [16].

With respect to the SF-36, moderate correlations were observed between the FRI and domains reflecting physical functioning and social participation, indicating parallel trends in Qol and functional status among individuals with low back pain. Weaker correlations were observed with the mental and emotional domains of the SF-36. This finding is expected, as the FRI was designed to assess pain-related functional disability rather than psychological constructs. Therefore, lower associations with mental health domains may reflect appropriate divergent validity rather than a limitation of the instrument. These findings are consistent with previous studies comparing the FRI with the SF-36 [7,15,33], and should also be interpreted in light of the ceiling effects observed in the Role Physical and Role Emotional domains, which are known to limit score variability [30].

The consistency of reliability and validity indices across multiple cultural adaptations suggests that the FRI captures a relatively stable construct of pain-related disability, minimally influenced by linguistic or contextual factors. This cross-cultural stability strengthens its suitability for international comparative studies and multicenter research.

Taken together, these findings support the methodological rigor and clinical interpretability of the Italian FRI in individuals with CLBP. By providing robust reliability estimates alongside measurement error quantification, the present study enhances the instrument’s utility not only as a descriptive disability measure but also as a decision-support tool within evidence-based rehabilitation pathways.

Notably, the results from the FRI on a group of people with Italian NP were similar to our results for CLBP (α = 0.88 vs. α = 0.92; NRS vs. VAS: 0.66 and 0.60; ODI vs. Neck Disability Index: 0.77 vs. 0.73, respectively), further supporting the applicability of the instrument across different spinal conditions within the Italian context [19].

This study has several limitations. First, the relatively small sample size may have limited the precision of the psychometric estimates, particularly reliability indices and correlation coefficients. Although the sample size exceeded the minimum threshold recommended for reliability analyses of PROMs, no formal power analysis was performed specifically for the ICC estimation. Future studies with larger samples could provide more precise reliability estimates. A second limitation is that structural validity was not formally assessed. Although previous studies on the FRI have explored its dimensional structure, confirmatory factor analysis or Rasch analysis were not performed in the present sample. Since structural validity represents an important measurement property, future studies should further investigate the factorial structure and dimensionality of the Italian version of the FRI. Third, a potential selection bias cannot be excluded, as participants were consecutively recruited from clinical settings, which may limit the representativeness of the broader population with LBP. Another limitation concerns the assessment of test–retest reliability. Although a short retest interval and the absence of treatment modifications were used to minimize clinical change, no formal external anchor (e.g., Global Rating of Change) was employed to confirm patient stability. Therefore, the ICC estimates should be interpreted with caution, and future studies should include explicit stability measures to strengthen reliability assessment. Fourth, the association between physical performance measures or clinical tests and the ability to detect spinal impairments was not examined, and further assessments are recommended. Fifth, other relevant psychometric properties, such as responsiveness and minimally important change, could not be evaluated due to the cross-sectional design, and longitudinal studies are therefore warranted. Finally, the findings are specific to individuals with CLBP, and further research is needed to extend generalizability to other LBP etiologies or pain durations. 

### Future Perspectives

Furthermore, while PROMs remain a cornerstone for assessing patient-perceived disability, contemporary research increasingly emphasizes the integration of objective biomechanical assessments. For instance, the use of inertial measurement units (IMUs) has shown promise in objectively quantifying functional impairments and gait alterations in lumbosacral pathologies [34]. Acknowledging these emerging sensor-based approaches could provide a more comprehensive characterization of spinal disorders by combining subjective disability scales with objective movement analysis.

## 5. Conclusions

The Italian version of the FRI demonstrated satisfactory reliability, interpretability, and construct validity in individuals with CLBP, consistent with findings from other language adaptations. These results suggest that the instrument may represent a useful tool for assessing pain-related functional disability in Italian-speaking individuals with CLBP. However, further studies are warranted to investigate additional measurement properties, including structural validity, responsiveness, and minimal important change, in order to strengthen its applicability in clinical and research settings.

## Figures and Tables

**Table 1:** Socio-demographic characteristics of the study population (n = 75).

Age (years), mean ± SD		48.71 ± 19.18
Gender (n)	Male	23 (30.7%)
	Female	52 (69.3%)
Marital status (n)	Married	41 (57.4%)
	Single	27 (36%)
	Divorced	3 (4%)
	Widowed	4 (5.3%)
Employment (n)	Students	12 (16.0%)
	Employed	30 (40.0%)
	Self-employed	7 (9.3%)
	Domestic works	7 (9.3%)
	Retired	18 (24%)
	Unemployed	1 (1.3%)
Education level (n)	Primary school	0 (0%)
	Middle school	5 (6.7%)
	High school	34 (45.3%)
	University	36 (48.0%)
Smokers (n)	Yes	11 (14.7%)
	No	64 (85.3%)
Alcohol (n)	Yes	13 (17.3%)
	No	62 (82.7%)
Physical activity (n)	Yes	46 (61.3%)
	No	29 (38.7%)
Comorbidities (n)	None	44 (58.7%)
	Cardiac	6 (8%)
	Respiratory	1 (1.3%)
	Gastro-intestinal	6 (8%)
	Renal	10 (13.3%)
	Headache	3 (4%)
Body Mass Index (kg/m^2^), mean ± SD		23.79 ± 3.58

SD: standard deviation; n: raw number; %: percentage.

**Table 2:** Distribution of FRI questionnaire, RMQ, ODI, NRS scores, and SF-36 domains.

	Mean	SD	25th%	50th%	75th%	Floor Effect [%]	Ceiling Effect [%]
FRI (0–100)	29.26	15.56	17.5	27.5	37.5	0	0
RMQ (0–24)	8.47	6.53	2	5	8	0	0
ODI (0–50)	7.48	5.36	3	7	12	0	0
NRS (0–10)	2.64	3.36	1	2	5	0	0
SF-36 Physical functioning (0–100)	73.87	24.68	60	80	95	0	0
SF-36 Physical role (0–100)	67.33	38.99	25	100	100	0	51
SF-36 Bodily pain (0–100)	58.83	20.74	41	52	74	0	0
SF-36 General health (0–100)	41.07	14.64	30	40	55	0	0
SF-36 Vitality (0–100)	51.53	17.28	45	50	65	0	0
SF-36 Social functioning (0–100)	70.83	20.06	62.50	75	87.50	0	0
SF-36 Emotional role (0–100)	68.44	39.47	33.33	100	100	0	53
SF-36 Mental Health (0–100)	61.87	18.37	48	64	76	0	0

FRI: Functional Rating Index; RMQ: Roland Morris Disability Questionnaire; ODI: Oswestry Disability Index; NRS: numerical rating scale; 36-Item Short-Form Health Survey.

**Table 3:** Construct validity between FRI and the other outcome measures.

Outcome Measures	FRI (r)	95% CI	*p* Value
RMQ	0.70	0.55–0.80	<0.001
ODI	0.74	0.61–0.83	<0.001
NRS	0.60	0.43–0.73	<0.001
SF-36 Physical Functioning	−0.38	−0.56–−0.17	<0.001
SF-36 Physical Role	−0.41	−0.58–−0.20	<0.001
SF-36 Bodily Pain	−0.58	−0.71–−0.40	<0.001
SF-36 General Health	−0.37	−0.55–−0.16	<0.001
SF-36 Vitality	−0.56	0.70–−0.38	<0.001
SF-36 Social Functioning	−0.48	−0.64–−0.29	<0.001
SF-36 Emotional Role	−0.27	−0.47–−0.04	0.020
SF-36 Mental Health	−0.26	−0.46–−0.03	0.025

r: Pearson coefficient correlation between FRI and other outcomes measures. RMQ: Roland Morris Disability Questionnaire; ODI: Oswestry Disability Index; NRS: numerical rating scale; SF-36: 36-Item Short-Form Health Survey; CI: Confidence Interval.

## Data Availability

The original contributions presented in this study are included in the article. Further inquiries can be directed to the corresponding author.
